# Chronic Arsenic Exposure and Oxidative Stress: *OGG1* Expression and Arsenic Exposure, Nail Selenium, and Skin Hyperkeratosis in Inner Mongolia

**DOI:** 10.1289/ehp.8723

**Published:** 2006-02-09

**Authors:** Jinyao Mo, Yajuan Xia, Timothy J. Wade, Michael Schmitt, X. Chris Le, Runhe Dang, Judy L. Mumford

**Affiliations:** 1 National Research Council, Washington, DC, USA; 2 Inner Mongolia Center for Endemic Disease Control and Research, Huhhot, Inner Mongolia, China; 3 National Health and Environmental Effects Research Laboratory, U.S. Environmental Protection Agency, Research Triangle Park, North Carolina, USA; 4 University of Alberta, Edmonton, Alberta, Canada

**Keywords:** arsenic, blood, humans, nail, *OGG1*, oxidative stress, selenium, skin hyperkeratosis

## Abstract

Arsenic, a human carcinogen, is known to induce oxidative damage to DNA. In this study we investigated oxidative stress and As exposure by determining gene expression of *OGG1*, which codes for an enzyme, 8-oxoguanine DNA glycosylase, involved in removing 8-oxoguanine in As-exposed individuals. Bayingnormen (Ba Men) residents in Inner Mongolia are chronically exposed to As via drinking water. Water, toenail, and blood samples were collected from 299 Ba Men residents exposed to 0.34–826 μg/L As. RNA was isolated from blood, and mRNA levels of *OGG1* were determined using real-time polymerase chain reaction. *OGG1* expression levels were linked to As concentrations in drinking water and nails, selenium concentrations in nails, and skin hyperkeratosis. *OGG1* expression was strongly associated with water As concentrations (*p* < 0.0001). Addition of the quadratic term significantly improved the fit compared with the linear model (*p* = 0.05). The maximal *OGG1* response was at the water As concentration of 149 μg/L. *OGG1* expression was also significantly associated with toenail As concentrations (*p* = 0.015) but inversely associated with nail Se concentrations (*p* = 0.0095). We found no significant differences in the As-induced *OGG1* expression due to sex, smoking, or age even though the oldest group showed the strongest *OGG1* response (*p* = 0.0001). *OGG1* expression showed a dose-dependent increased risk of skin hyperkeratosis in males (trend analysis, *p* = 0.02), but the trend was not statistically significant in females. The results from this study provide a linkage between oxidative stress and As exposure in humans. *OGG1* expression may be useful as a biomarker for assessing oxidative stress from As exposure.

Arsenic is ubiquitous in the environment. Human exposure to As has been associated with cancers (lung, bladder, skin) and other chronic diseases such as dermal, cardiovascular, neurologic, and diabetic effects (Abernathy et al. 2003; Yoshida et al. 2004). The major route of human exposure for inorganic As is through consumption of contaminated drinking water, especially in some regions such as India, Bangladesh, Taiwan, and China (Ahsan et al. 2000; Chen et al. 2005; Mazumder et al.1998; Xia and Liu 2004). In 2001, the U.S. Environmental Protection Agency (EPA) adopted an As standard of 10 μg/L in drinking water (U.S. EPA 2001). There are still great uncertainties on health effects of As at low doses. Research is needed to investigate the mode of action for As effects and assess human health effects of As at low concentrations using biologic markers.

Arsenic is known for generation of reactive oxygen species (ROS). Studies have shown that exposure to As generates superoxide, hydrogen peroxide, and hydroxyl radicals in keratinocyte cells *in vitro* (Shi et al. 2004). Mice treated with arsenite show evidence of free radicals in the liver (Liu SX et al. 2001). Chronic exposure to As in a contaminated area of Inner Mongolia increased lipid peroxide concentrations in serum (Pi et al. 2002). Increased reactive oxidants and decreased antioxidants in blood were reported in a Taiwanese population exposed to As (Wu et al. 2001). ROS can interact with DNA to produce damage including DNA breaks, deletions, and hydroxylation of 2′-deoxyguanosine. Oxidation at the C8 position of guanine (8-oxoguanine) may cause mutation due to its potential to mispair with adenine during DNA replication. Arsenic has been shown to increase urinary 8-hydroxydeoxyguanine levels in animal and human studies (Fujino et al. 2005; Wei et al. 2002; Yamauchi et al. 2004).

DNA is susceptible to oxidative damage such as oxidation of DNA bases. Thus, the capacity and efficiency of the repair enzymes are of great importance for cancer risk assessment in humans. Environmental contaminants that induce DNA damage may also increase gene expression of DNA repair genes (Vogel et al. 2002). *OGG1* encodes 8-oxoguanine DNA glycosylase, which is involved in base excision repair of 8-oxoguanine caused by the effects of ROS on DNA (Memisoglu and Samson 2000). Induction of *OGG1* expression has been shown to correlate with the repair capacity of 8-oxoguanine (Kondo et al. 2000). Animal studies show the induction of *OGG1* expression in rat lungs after instillation of diesel exhaust particles (Tsurudome et al. 1999). An *in vitro* study in human colorectal carcinoma cells showed that when cells were treated with methylmethane sulfonate (a DNA-alkylating agent), OGG1 mRNA levels increased significantly, and the increased levels of *OGG1* expression were correlated with increase in enzyme activity (Lee et al. 2004). These studies suggested that mRNA expression of the DNA repair gene *OGG1* may be useful in assessing oxidative stress induced by the toxicants and can serve as a biomarker of exposure to reactive oxygen radicals. No studies have been reported on the effects of chronic As exposure on *OGG1* gene expression in humans. Monitoring expression of *OGG1* may be of great value in investigating the oxidative stress induced by As.

Bayingnormen (Ba Men) is a region located on the Hetao Plain, north of the Huang He River in western Inner Mongolia, China (Guo et al. 2003). In Ba Men, more than 300,000 people have been chronically exposed to As (Ma et al. 1999). Arsenic is naturally occurring in groundwater in Ba Men, especially in the three counties of Hangjin Hou, Lin He, and Wu Yuan. The location of Ba Men and the three counties is shown in Figure 1. Ba Men residents reported that As-containing pesticides were not used. The residents in Ba Men, who are mostly farmers, have been exposed to a wide range of As levels (ranging from below detection limit to 1.8 mg/L), mainly via drinking water from artesian wells, for > 20 years. Health effects at multiple end points, including dermal, neurologic, cardiovascular, and peripheral vascular effects, have been reported (Ma et al. 1999). More than 80% of the families own individual wells, and it is possible to assess As exposure on an individual basis. The Ba Men region is well known in China for its abundance of agriculture. The Ba Men residents produce wheat, corn, sunflower seeds, vegetables, and fruits and also raise domestic animals for commercial purposes and their own consumption. This population provides good opportunities to investigate health effects of chronic As exposure via drinking water and to evaluate the utility of biomarkers for assessing As effects at low doses.

In this study we investigate the association of human OGG1 mRNA levels in blood and chronic As exposures in the Ba Men population. Because selenium has been reported to affect the toxicity of As (Patrick 2003), we also investigated the relationship between *OGG1* expression and nail Se levels. In addition, we linked *OGG1* expression to skin hyperkeratosis, which is a typical skin lesion from chronic As exposure in humans (Ahsan et al. 2000).

## Materials and Methods

### Study subjects

**Figure 1:**
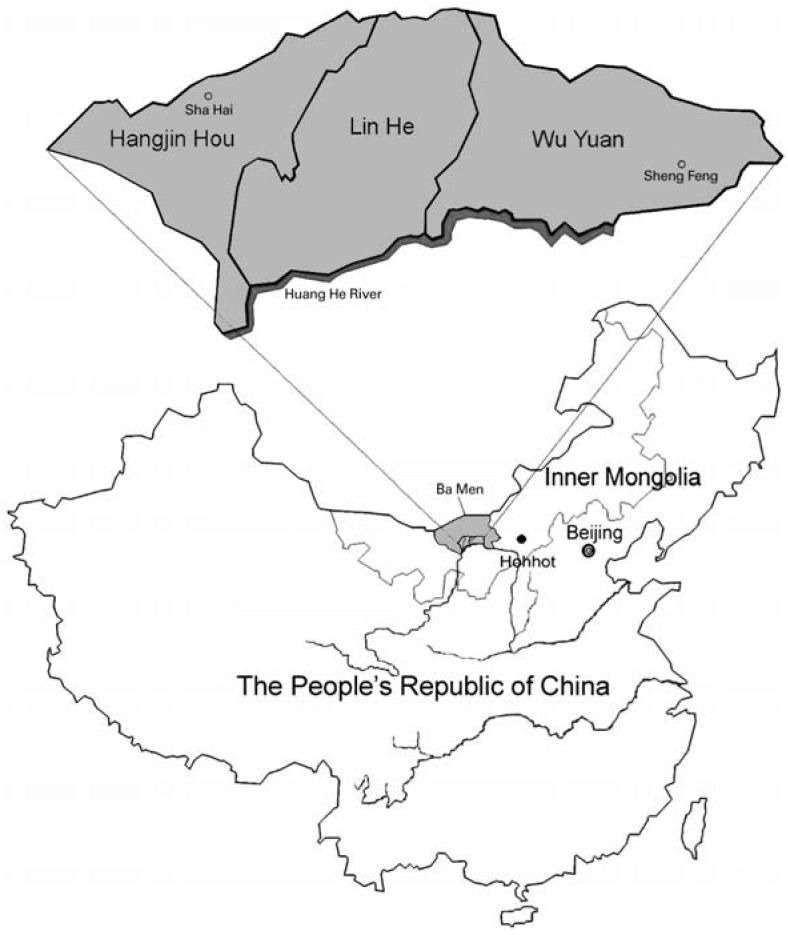
Map of three counties in Ba Men where residents have been chronically exposed to As via drinking water. The study sites are located at Sha Hai Village in Hangjin Hou County and Sheng Feng Village in Wu Yuan County.

The study subjects included a total of 299 Ba Men residents from the sub-villages of Wulan, Jianshe, Fengchan, and Xinyao located in Sha Hai Village, Hangjin Hou County, and the subvillages of Miaohao and Xigelian, located in Sheng Feng Village, Wu Yuan County (Figure 1). These subvillages were selected because most residents have been exposed to a wide range of As levels in drinking water from the artesian wells for > 10 years. Before subject selection, well-water samples of the homes from these subvillages were collected and analyzed for As concentrations. The study subjects were selected according to the criteria set for the study design focusing on the As effects on *OGG1* expression at low doses (≤200 μg/L). The criteria included *a*) approximately 70% of subjects with As exposure levels from nondetectable to 200 μg/L and 30% with As exposure levels > 200 μg/L, with exposures of at least 5 years; *b*) approximately equal numbers of males and females and equal numbers of smokers and nonsmokers; and *c*) age ranging from 11 to 60 years. Questionnaires were administered to all participants to obtain demographic information, history of well use, diet, smoking, occupation, pesticide use, and medical information. Diagnosis for skin hyperkeratosis, depigmentation, and hyperpigmentation was based on the China national standards for diagnosis of arsenicosis (People’s Republic of China 2000). Skin hyperkeratosis is the presence of benign wart-like growths on the skin. Skin hyperpigmentation is the presence of dark pigmentation, and depigmentation is the presence of pale or light brown color on the skin.

This study was conducted according to the recommendations of the Declaration of Helsinki (World Medical Association 1989) for international health research. All subjects gave written informed consent to participate in this study. The research protocol met the requirements for protection of human subject certification and was approved by the U.S. EPA.

### Water collection and analysis

Samples of drinking water from wells were collected from all subjects’ homes in acid-washed tubes, transported to the United States on ice packs, stored at –80°C, and analyzed for total As using inductively coupled plasma mass spectrometry (ICPMS) as described previously (Gong et al. 2006). Briefly, the frozen water samples were thawed at room temperature and then acidified to contain 1% HNO_3_. The acidification took place in the original sample tubes to account for any possible adsorption of As on the walls of the sample tubes. The pH of the samples after acidification was 1.4–1.6. An aliquot (1–2 mL) of each acidified sample was diluted 5- to 10-fold with 1% HNO_3_ for subsequent As analysis using a PerkinElmer 6100 DRC^plus^ inductively coupled plasma mass spectrometer (PerkinElmer/Sciex, Concord, Ontario, Canada). Similarly, As calibration standard solutions were also prepared in 1% HNO_3_. For quality control, a standard reference material (SRM1643d water; National Institute of Standards and Technology, Gaithersburg, MD, USA) was periodically analyzed. The result from 44 analyses of the SRM1643d (certified value, 56.0 μg/L) was 57.7 ± 2.6 μg/L. Water samples spiked with As and method blanks were also periodically analyzed. The detection limit by ICPMS is 0.1 μg/L.

### Toenail collection and analysis

Nail samples from all 10 toes were collected from each subject, stored in plastic bags, and shipped back to the United States. The nail samples were first cleaned by sonication in HPLC-grade water. The water was removed, and acetone was added to remove the organic contaminants from the nail surface (Schmitt et al. 2005). Nail samples were analyzed for As and Se content by instrumental neutron activation analysis (INAA) at the Nuclear Services Department, North Carolina State University (Raleigh, NC, USA) (Heydorn 1984). We used a nuclear reactor (1 MW PULSTAR; American Machine and Foundry, New York, NY, USA) as the neutron source for the irradiation of toenail samples. During irradiation, natural stable isotopes of As or Se contained in the samples were transformed into radioactive nuclides by neutron capture. Activated trace radioisotopes of As (^76^As) or Se (^75^Se) were analyzed and quantified using high-efficiency gamma spectrometry systems. Traceable standards, standard reference material, method blanks, and sample duplicates were processed along with the samples for quality assurance. The levels of the detection limit by INAA were 0.012 μg/g for As and 0.064 μg/g for Se.

### Blood collection, RNA isolation, and cDNA synthesis

Peripheral blood samples were collected in PAXgene blood RNA tubes (Qiagen, Valencia, CA, USA), placed on ice, and then stored at –40°C. The PAXgene blood tubes contain reagents to stabilize RNA in blood. The blood samples were transported to the United States via air on ice packs and stored at –80°C until RNA isolation. Total RNA was isolated using a PAXgene blood RNA kit according to the manufacturer’s instructions. cDNA was synthesized (50 μL total volume) from 500 ng of total RNA using Superscript III reverse transcriptase (Invitrogen, Carlsbad, CA, USA).

### OGG1 mRNA assay

An aliquot of 2 μL cDNA was used for each assay. Real-time polymerase chain reaction (PCR) was performed to compare OGG1 mRNA levels on an ABI 7700 Sequence Detector System (Applied Biosystems, Foster City, CA, USA). The real-time PCR reaction mixture contained Universal Mastermix (Applied Biosystems), 100 nM Taqman probe, and 200 nM primers. Primers and probes (assay ID Hs00162669_m1) were obtained from Applied Biosystems. The primers span exon 3 and exon 4 junctions in the *OGG1* cDNA sequence. OGG1 mRNA levels were determined by a relative standard curve method (Applied Biosystems 2001). Human skin keratinocyte (HaCaT) RNA was used to construct standard curves. The mRNA quantities of the tested samples were determined from the standard curves and were normalized to the endogenous reference, β-actin levels. Each sample was assayed in duplicate, and the mean value of the duplicates was used for data analysis. Some selected samples (5%) were assayed twice to check the reproducibility (> 95%). All of the samples were tested in a blind fashion regarding the As exposure for the study subjects.

### OGG1 *assay controls.*

After comparing 11 candidate genes for the endogenous control in the assay, we selected β-actin for this study. β-Actin expression showed the least variation among the genes examined (data not shown). OGG1 mRNA levels were normalized by the levels of β-actin, which was measured in the same tube of each OGG1 mRNA measurement. Distilled water and blood total RNA were used as the negative controls. For a positive control, we used samples of cDNA from HaCaT exposed to As *in vitro* that were positive for *OGG1* expression. For a human control, we used a composite cDNA sample from 10 pooled subjects in this study. All controls were run simultaneously with the test samples throughout the experiments. The coefficients of variance for the repeated measurements for the composite human blood and the HaCaT positive controls were 5% and 7%, respectively.

### Statistical analysis

Bivariate associations between categorical variables were evaluated using chi-square tests for homogeneity. We used linear regression models and Spearman and Pearson correlation coefficients to evaluate the association between continuous dependent variables (*OGG1* expression) and independent predictor variables. Nonlinearity in the relationship between *OGG1* expression and water As was assessed by using cubic spline and quadratic transformations of the As measure. We used logistic regression models to evaluate the association between binary dependent variables (skin hyperkeratosis) and independent predictor variables. Interaction terms between key covariates (age, sex, smoking) and predictors were included in regression models. For logistic models, likelihood ratio tests were used to determine the importance of the interaction terms, and for linear models, the significance of the interaction coefficient was evaluated using the Wald test. Multivariate models were used to control for the effect of potential confounding factors. Variables included in the multivariate model were selected based on our understanding or belief that these variables could bias the *OGG1* expression and As relationship, or if they were strongly associated with either the As exposure or the outcome (*OGG1* expression) in the bivariate analyses. We conducted separate (stratified) analyses by age category, sex, and smoking status to evaluate whether the relationship between *OGG1* expression and As differed in the subgroups (i.e., interaction) and whether there was a particular sensitivity in any of the subgroups. We assessed the importance of the interactions by evaluating the strength and significance of interaction terms in the model and by plotting and qualitatively examining the differences in the *OGG1* expression–As exposure relationship.

Continuous dependent variables were transformed to a log scale when they departed from normality as observed by quantile–quantile plots, Shapiro-Wilk tests for normality, and histograms. In many cases, transforming to a log scale made the dependent variables approximately normally distributed. Water As concentrations were also transformed to a log scale because the natural distribution of this variable was highly skewed. After transformation, the water As measure, although considerably less skewed, showed departure from normality. For correlations between two normally distributed variables, Pearson correlation coefficients were reported. When one or both of the variables displayed departures from normality, the Spearman rank-correlation coefficient was reported. Statistical analyses were conducted using SAS (SAS Institute 2001) and Stata (StataCorp 2005) software.

## Results

### Study subjects

**Figure 2:**
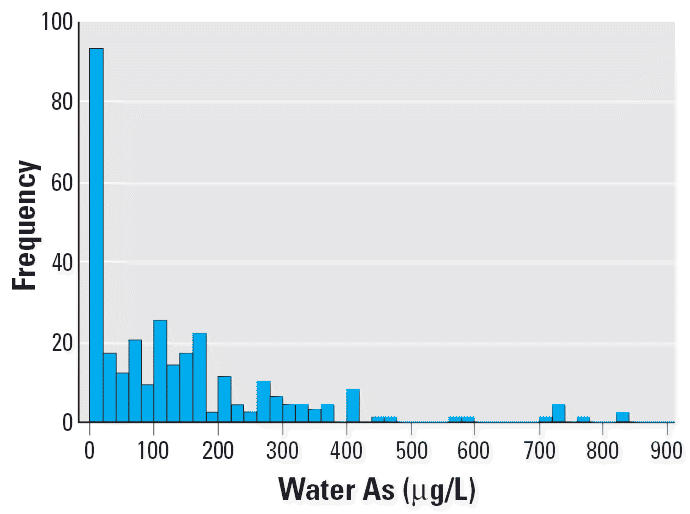
Frequency distribution of study subjects according to As concentrations of well water (*n* = 299).

Table 1 shows the characteristics of the study subjects. The study subjects, mostly farmers, have been exposed to As via drinking water with concentrations ranging from 0.34 to 826 μg/L, with a mean exposure time of 14 years. Figure 2 shows the frequency distribution of study subjects according to the As concentrations of drinking water. Seventy-seven percent of subjects have been exposed to As at 0.34–200 μg/L, and 23% of subjects were exposed to 201–826 μg/L. Among the subjects, 154 were male and 145 were female. The ages ranged from 11 to 60 years (median, 38 years). Most subjects (77%) received no more than a ninth grade education. Thirty-four percent were smokers, and most subjects (81%) did not drink alcohol or drank less than twice a week. Forty-nine percent of subjects reported exposure to pesticides in the past 5 years. Most subjects (99%) reported eating meat, dairy products, and green vegetables often, suggesting that they have a good diet. Four percent of the subjects took vitamins regularly. Because the study subjects live in the remote inland villages in Inner Mongolia, seafood (from salt water) is not readily available. The residents living in the area occasionally consume fish from the Huang He River. A previous report showed that the As levels of the water from the Huang He River are below the Chinese As standard for drinking water (< 50 μg/L) (Ma et al. 1999).

### Water As versus nail As

**Figure 3:**
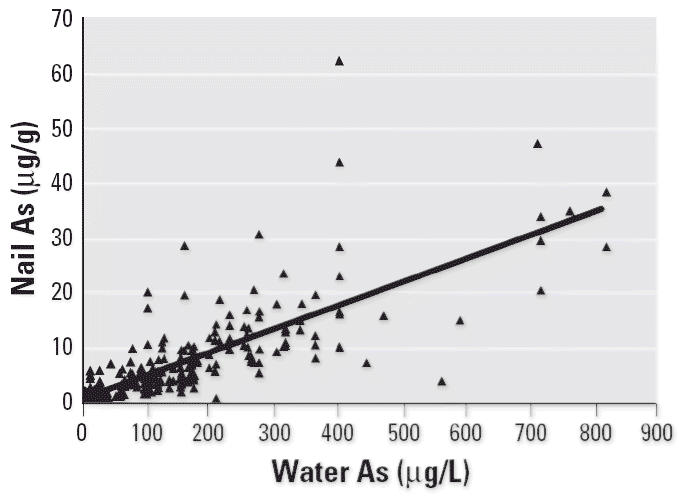
High correlation between nail and water As concentrations (*n* = 299; Spearman *r* = 0.8816; *p* < 0.0001; nail As = 1.135 + 0.04277 × water As).

Toenail and drinking-water As concentrations showed a strong positive correlation (Spearman *r* = 0.8816, *p* < 0.0001), as shown in Figure 3. This finding substantiated that the main source of As exposure for these individuals was drinking water.

### OGG1 *expression versus water As.*

**Figure 4:**
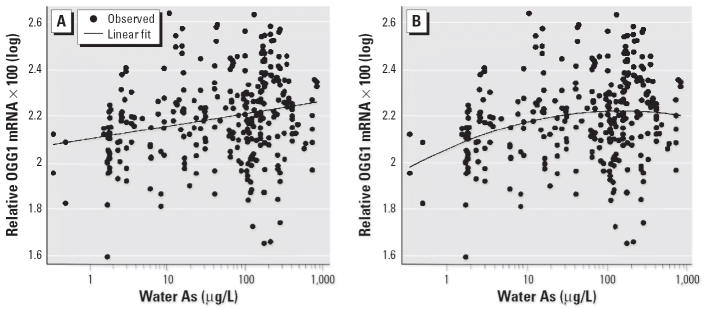
Association between OGG1 mRNA levels and water As concentrations (*n* = 299) by linear (*A*) and quadratic (*B*) modeling. OGG1 mRNA levels and water As for the subjects were all normalized to β-actin levels, multiplied by 100, and log transformed. (*A*) OGG1 mRNA concentrations were positively associated with water As concentrations using the linear model [Spearman *r* = 0.24; *p* < 0.0001; OGG1 × 100(log) = 2.10 + 0.054 × water As(log)]. (*B*) OGG1 mRNA concentrations were positively associated with water As concentrations using the quadratic model [adjusted *r* = 0.24; *p* < 0.0001; OGG1 × 100(log) = 2.05 + 0.15 × water As(log) – 0.035 × water As(log)^2^].

Induction of *OGG1* expression was positively associated with As exposure via drinking water for all subjects using the linear model (*p* < 0.0001) (Figure 4A). A model that included a quadratic term for water As was also statistically significant (*p* < 0.0001) (Figure 4B). Addition of the quadratic term significantly improved the fit compared with the linear model (*p* = 0.05). Plots of the cubic spline transformation (not shown) confirmed that an approximate quadratic relationship was appropriate. By solving the quadratic equation for the slope of the derivative of the function equal to zero, it was determined that the maximum *OGG1* response was observed at an As exposure of approximately 149 μg/L, and then the response leveled off or decreased at higher concentrations.

**Figure 5:**
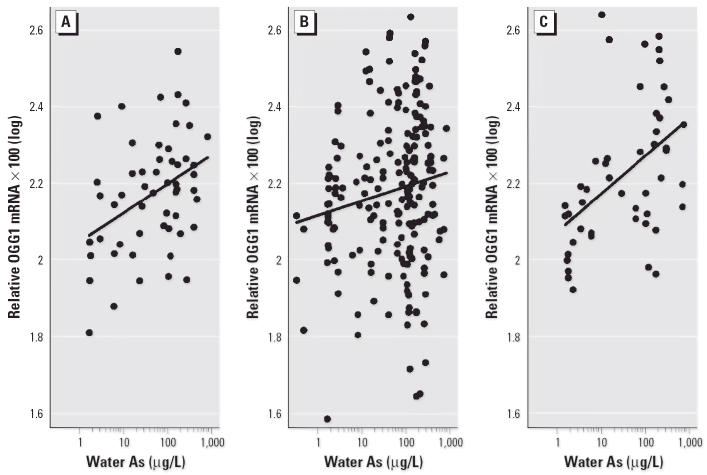
Effect of age on As-induced *OGG1* expression for three age groups: (*A*) 11–18 years of age [*n* = 54; Spearman *r* = 0.37; *p* = 0.0062; OGG1 × 100(log) = 2.05 + 0.075 × water As(log)]; (*B*) 19–50 years of age [*n* = 196; Spearman *r* = 0.16; *p* = 0.03; OGG1 × 100(log) = 2.12 + 0.038 × water As(log)]; (*C*) 51–60 years of age [*n* = 48; Spearman *r* = 0.54; *p* = 0.0001; OGG1 × 100(log) = 2.07 + 0.099 × water As(log)].

No significant difference in the association between As and *OGG1* expression was observed between males and females (*p* = 0.8 for interaction) or between nonsmokers and smokers (*p* = 0.83 for interaction), although the nonsmokers showed slightly higher levels of *OGG1* expression. *OGG1* expression levels increased with water As concentrations in all age groups (Figure 5). The interaction between age and the effects of As concentration on *OGG1* expression was not statistically significant (*p* = 0.18) even though the association appeared to be strongest in the oldest group (51–60 years of age; *p* = 0.0001) followed by the youngest group (11–18 years of age; *p* = 0.0062).

### OGG1 *expression versus nail As and Se.*

**Figure 6:**
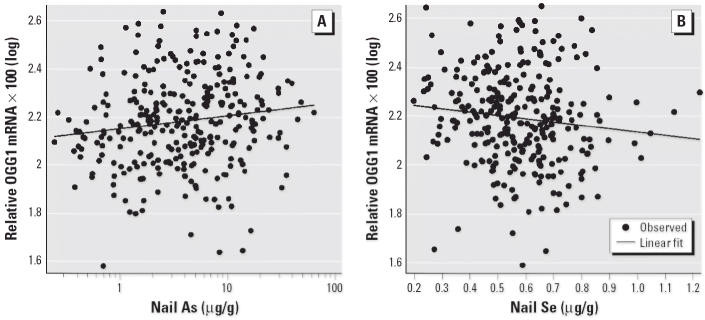
Association between *OGG1* expression and nail As and nail Se concentrations. (*A*) Positive association between *OGG1* expression and nail As concentration [*n* = 299; Pearson *r* = 0.14; *p* = 0.015; OGG1 × 100(log) = 2.15 + 0.054 × nail As(log)]. (*B*) Negative association between *OGG1* expression and nail Se concentrations [*n* = 288; Spearman *r* = –0.16; *p* = 0.0095; OGG1 × 100(log) = 2.26 – 1.35 × nail Se].

*OGG1* expression was positively associated with nail As concentrations (*p* = 0.015), as shown in Figure 6A, but was inversely associated with nail Se concentrations (*p* = 0.0095), as shown in Figure 6B.

### OGG1 *expression versus dermal effects.*

Thirty-one percent of the subjects showed skin hyperkeratosis, 4% skin hyperpigmentation, and 11% skin depigmentation (Table 1). Skin hyperkeratosis occurred with similar frequency by age category (*p* = 0.2, chi-square test) and sex (*p* = 0.86, chi-square test). *OGG1* expression in males showed a strong dose-dependent increased risk of skin hyperkeratosis (trend analysis *p* = 0.02) in all four quartile groups (Table 2). Males in the highest *OGG1* expression category (*OGG1*, 2.02–4.37) experienced hyperkeratosis nearly three times more frequently than those in the lowest category [odds ratio (OR) = 2.98, *p* = 0.047]. In females, the association between hyper-keratosis and *OGG1* expression showed some evidence of a dose-dependent increased risk in the first three quartile groups, although the trend was not statistically significant (*p* = 0.22). As expected, there was a significant dose–response relationship between the risk of skin hyperkeratosis and water As concentration (*p* < 0.0001 for trend analysis) (Table 3). Higher *OGG1* expression did not indicate a significant increase in risk of skin depigmentation and hyperpigmentation (*p* = 0.895).

## Discussion

In this study we investigated the effects of As on the expression of *OGG1* in blood in a human population and linked *OGG1* gene expression to nail As and Se. We also assessed the relationship between *OGG1* gene expression and skin hyperkeratosis. This is the first study showing that OGG1 mRNA expression in blood was associated with As levels in drinking water in a human population. In addition, we investigated other factors that may affect *OGG1* expression. There were no significant differences in *OGG1* induction due to sex or tobacco smoking. There was some evidence of a difference in the relationship between As and *OGG1* induction by age group (with the strongest effect in the oldest group), but the interaction was not statistically significant. We also found that pesticide exposure increased *OGG1* gene expression (*p* = 0.04, chi-square test; data not shown), but other demographic factors such as education, occupation type, and consumption of meat, dairy, and freshwater seafood were not associated with *OGG1* gene expression.

To assess the relationship between *OGG1* gene expression and As exposure, we used both a linear model and a quadratic (nonlinear) model. The quadratic model showed that the maximal *OGG1* response was at an As exposure level of 149 μg/L and that *OGG1* expression leveled off or decreased when As exposure was higher than this concentration. The finding of this adaptive response of increasing expression of the DNA base excision repair gene *OGG1* is in agreement with reports that at low doses (0.1–1 μM) As induces significant up-regulation of adaptive responses, such as enhanced cell proliferation, telomerase activity, and base excision repair (DNA polymerase β, DNA ligase I), whereas at high concentrations (> 1 μM) these processes are down-regulated in cultured human cells (Simeonova et al. 2000; Snow et al. 2005; Zhang et al. 2003).

The mechanisms of As carcinogenicity and other chronic effects in humans are not completely understood. The major modes of action by As that have been reported are DNA damage, oxidative stress, chromosome aberrations, apoptosis, DNA methylation, signal transduction, DNA repair, and cell proliferation (Chen et al. 2005; Hughes 2002; Rossman 2003). Oxidative damage caused by ROS may directly or indirectly influence all of these actions underlying carcinogenesis and other toxic effects of As (Liu SX et al. 2001; Shi et al. 2004). Wu et al. (2001) found an association of blood As levels with increased reactive oxidants and decreased antioxidant capacity in the residents of Taiwan. A study conducted in Inner Mongolia showed that As-exposed individuals have higher serum lipid peroxide and lower nonprotein sulfhydryl levels, indicating an association of As exposure and oxidative stress (Pi et al. 2002). Urinary 8-hydroxy-2′-deoxyguanosine levels increased both in Japanese patients with acute As poisoning and in Chinese individuals chronically exposed via drinking water (Yamauchi et al. 2004). Our study results on *OGG1* gene expression further substantiate these studies. In the present study, we observed increased expression of *OGG1* associated with chronic As exposure from drinking water. Because OGG1 is the main enzyme responsible for removing 8-oxyguanine from DNA, this study supports previous reports that As increases the level of oxidative damage to DNA. A study using cDNA microarrays also showed elevated *OGG1* gene expression in the liver of mice treated with inorganic arsenicals (Liu J et al. 2001). That finding is in agreement with our findings here.

There are some possible mechanisms to explain the As-induced *OGG1* gene expression that we found in this study. The *OGG1* promoter region contains transcription factor binding sites for Nrf2 and AP-1 (Dhenaut et al. 2000; Venugopal and Jaiswal 1998). *OGG1* expression may be modulated by these two transcription factors. The Nrf2 transcription factor, which regulates the antioxidant response, can be activated by hydrogen peroxide generated by arsenite in human keratinocytes (Pi et al. 2003). Because the *OGG1* promoter region contains the Nrf2 binding site, *OGG1* gene expression may be modulated by oxidative stress resulting from exposure to As in this population. AP-1 is a DNA-binding protein composed of the Jun and Fos proteins (Cavigelli et al. 1996), and As has been shown to induce the expression of the protooncogenes c-*fos* and c-*jun* and AP-1 transcriptional activity. Studies in rat lung have shown that arsenite enhances the binding of AP-1 to DNA and induces stress protein expression, including heat-shock proteins HSP32 and HSP72 (Wijeweera et al. 2001). Therefore, it is possible that induction of Nrf2 and AP-1 by As may increase DNA binding activity of these transcription factors in As-exposed individuals and thus increase the expression of the oxidative stress gene *OGG1* found in this study.

Selenium along with other antioxidants, such as glutathione, vitamins, and methionine, have been shown to reduce oxidative damage caused by As toxicity (Mayne 2003). Selenium decreases As toxicity in animals and is also useful as a therapy for As detoxification in humans (Rabbani et al. 2003; Yang et al. 2002). Selenium is able to complex with glutathione and As to form a compound that can be excreted through the bile (Patrick 2003). In the present study, the levels of Se in toenails were inversely correlated with *OGG1* expression levels in blood, which further substantiated the role of As-induced oxidative stress and its reduction by Se in humans. With regard to dermal effects, increased *OGG1* expression showed increased risk of skin hyperkeratosis in males, but the trend was not statistically significant in females. The reason for the difference in sex is unknown or may be due to the small number of female subjects in the highest *OGG1* expression quartile.

## Conclusion

This study is the first report on the effects of chronic As exposure on the expression of *OGG1* in a human population. *OGG1* gene expression was positively associated with water and nail As concentrations but was inversely associated with nail Se concentrations. In addition, an increase in *OGG1* expression was associated with an increased risk of skin hyperkeratosis in males induced by chronic exposure to As. This study provides a linkage between oxidative stress and As exposure in humans. Because oxidative stress is a proposed mechanism underlying As carcinogenesis and other chronic toxic effects, these results can shed some light on the possible mode of action for As. *OGG1* gene expression may be useful as a biomarker to assess oxidative stress from chronic exposure to As in humans.

## Figures and Tables

**Table 1:** Study subjects’ characteristics.

Characteristic	No. (%)
Sex	
Male	154 (52)
Female	145 (48)
Age (years)	
11–18	54 (18)
19–50	196 (66)
51–60	48 (16)
Tobacco smoking	
Nonsmokers	199 (66)
Smokers	100 (34)
Education	
None	49 (16)
Elementary	92 (31)
Junior high school	139 (46)
High school	17 (6)
College or above	2 (< 1)
Alcohol	
At least twice a week	56 (19)
Less than twice a week	243 (81)
Occupation	
Farmer	234 (79)
Manufacturing	1 (< 1)
Other	63 (21)
Pesticide exposure in last 5 years	
Yes	146 (49)
No	153 (51)
Eat meat or dairy	
Never	0 (0)
Occasionally^a^	2 (< 1)
Often^b^	295 (99)
Eat freshwater fish	
Never	2 (< 1)
Occasionally	284 (96)
Often	11 (4)
Eat green vegetables	
Never	0 (0)
Occasionally	4 (1)
Often	293 (99)
Take vitamins regularly	
Yes	11 (4)
No	285 (96)
Skin hyperkeratosis	
Yes	91 (31)
No	207 (69)
Skin hyperpigmentation	
Yes	11 (4)
No	288 (96)
Skin depigmentation	
Yes	34 (11)
No	265 (89)

The total number of subjects is 299; some information was missing for some subjects.

a One to five times per month.

b More than five times per month.

**Table 2:** ORs for relationships between *OGG1* expression and skin hyperkeratosis.

	Skin hyperkeratosis				
*OGG1* expression	Absent	Present	OR (95% CI)^a^	*p*-Value	Trend *p*-value
All subjects					
0.38–1.17	56	18	1.00 (reference)		
1.17–1.53	54	21	1.33 (0.61–2.88)	0.470	
1.54–2.01	47	28	2.21 (1.03–4.75)	0.037	0.054^b^
2.02–4.37	50	24	1.69 (0.78–3.66)	0.180	0.099^c^
Male^d^					
0.38–1.17	32	9	1.00 (reference)		
1.17–1.53	33	9	1.06 (0.35–3.20)	0.92	
1.54–2.01	22	13	2.73 (0.92–8.10)	0.071	0.08
2.02–4.37	20	15	2.98 (1.02–8.75)	0.047	0.02
Female					
0.38–1.17	24	9	1.00 (reference)		
1.17–1.53	21	12	1.71 (0.57 –5.15)	0.34	
1.54–2.01	25	15	1.83 (0.60–5.59)	0.29	0.22
2.02–4.37	30	9	0.95 (0.30–3.01)	0.93	0.92

a Data shown are OR (95% CI) adjusted for sex, age, smoking, alcohol use, and pesticide use.

b *p*-Value for trend across first three quartiles of *OGG1* groups by Wald test.

c *p*-Value for trend across all four quartile categories by Wald test.

d Skin hyperkeratosis status was not ascertained for one male subject.

**Table 3:** ORs for relationship between water As concentrations and skin hyperkeratosis.

As concentration (μg/L)	Total^a^ (skin hyperkaratosis)^b^	OR (95% CI)^c^	*p*-Value
0.34–10	69 (11)	1.00	—
11–50	51 (14)	1.977 (0.777–5.029)	0.15241
51–100	30 (9)	2.477 (0.812–7.556)	0.11088
101–200	80 (24)	2.666 (1.140–6.231)	0.02364
201–300	33 (16)	5.180 (1.914–14.020)	0.00120
301–826	35 (17)	6.951 (2.551–18.938)	0.00015

*p* < 0.0001 for trend across all categories.

a Total number of subjects in this category; one missing value for As measure.

b Number of subjects with skin hyperkeratosis.

c Data shown are OR (95% CI) adjusted for sex, age, smoking, alcohol use, and pesticide use.
